# Childhood and family influences on body mass index in early adulthood: findings from the Ontario Child Health Study

**DOI:** 10.1186/1471-2458-12-755

**Published:** 2012-09-09

**Authors:** Andrea Gonzalez, Michael H Boyle, Katholiki Georgiades, Laura Duncan, Leslie R Atkinson, Harriet L MacMillan

**Affiliations:** 1 McMaster University, Department of Psychiatry and Behavioral Neuroscience, Offord Centre for Child Studies, 1280 Main Street West, Chedoke Site, Patterson Building, Hamilton, ON L8S 3K1, Canada; 2 Ryerson University, Department of Psychology, 350 Victoria Street, Toronto, ON, M5B 2 K3, Canada

**Keywords:** Family and child level risk factors, Body mass index

## Abstract

**Background:**

Overweight and obesity are steadily increasing worldwide with the greatest prevalence occurring in high-income countries. Many factors influence body mass index (BMI); however multiple influences assessed in families and individuals are rarely studied together in a prospective design. Our objective was to model the impact of multiple influences at the child (low birth weight, history of maltreatment, a history of childhood mental and physical conditions, and school difficulties) and family level (parental income and education, parental mental and physical health, and family functioning) on BMI in early adulthood.

**Methods:**

We used data from the Ontario Child Health Study, a prospective, population-based study of 3,294 children (ages 4–16 years) enrolled in 1983 and followed up in 2001 (N = 1,928; ages 21–35 years). Using multilevel models, we tested the association between family and child-level variables and adult BMI after controlling for sociodemographic variables and health status in early adulthood.

**Results:**

At the child level, presence of psychiatric disorder and school difficulties were related to higher BMI in early adulthood. At the family level, receipt of social assistance was associated with higher BMI, whereas family functioning, having immigrant parents and higher levels of parental education were associated with lower BMI. We found that gender moderated the effect of two risk factors on BMI: receipt of social assistance and presence of a medical condition in childhood. In females, but not in males, the presence of these risk factors was associated with higher BMI in early adulthood.

**Conclusion:**

Overall, these findings indicate that childhood risk factors associated with higher BMI in early adulthood are multi-faceted and long-lasting. These findings highlight the need for preventive interventions to be implemented at the family level in childhood.

## Background

Excess body weight is associated with numerous adverse health consequences including coronary heart disease, type II diabetes and cancer, among others [1,2]. In 2008, an estimated 1.46 billion adults worldwide were classified as overweight; of these, 502 million were obese [3]. These numbers are steadily increasing with the greatest prevalence of obesity occurring in high-income countries such as the United States and Canada. Obesity and its associated health complications have a significant economic impact on healthcare with annual national costs estimated at $4.6 to $7.1 billion in Canada [2], and $92.6 billion in the U.S. [4].

Obesity is a multifactorial condition influenced by diverse factors operating across the lifespan. Various family-level factors, as well as individual-level characteristics, have been identified as potential determinants of BMI in childhood and adulthood. Several prospective studies highlight the importance of childhood socioeconomic disadvantage as a major predictor of obesity [5-8]. For example, Power et al. (2003) showed that family SES in early childhood (birth to age 7) was significantly associated with obesity at age 33; this finding was not explained by parental BMI or the individual’s own education. These prospective studies illustrate that childhood SES has long-lasting effects that are not easily reversed by changes in SES occurring in adulthood [6]. Other family indicators, closely linked to SES, have been identified as risk factors for obesity in childhood. Children from single parent households have significantly higher BMIs compared to those from dual parent households [9]. In addition, parental educational attainment is inversely associated with adulthood BMI [10].

At an individual level, birth weight, as a crude estimate of in utero environment, is related to elevated BMI in childhood [11] and adulthood [12,13]. Various childhood psychosocial risk factors are also associated with elevated BMI in childhood and adulthood. In particular, diagnoses of depression, anxiety and conduct disorders in childhood and adolescence are related to increased BMI in adulthood [14-17]. Furthermore, obesity in young adulthood has been associated with behavioral problems exhibited at ages 5 to 14 years [18,19]. Individuals with a history of childhood sexual abuse [20] and physical abuse [21-23] are more likely to be overweight or obese later in life. Finally, there is some evidence to suggest that childhood learning difficulties, below average scholastic proficiency and having received special education are risk factors for obesity in young adulthood [24,25].

There is considerable evidence pointing to the importance of early life factors in the development of obesity in children and adults; however, the family as a contextual unit is rarely studied [26]. The family environment is considered key in the development of obesity [27], yet few studies have prospectively examined the impact of multiple family and childhood risks on BMI in early adulthood. We consider risk factors occurring at the child- and family levels simultaneously. In this study, we assessed a set of risk and protective variables, including prenatal risk (with low birth weight serving as marker), risk integral to the child (such as psychiatric disorder, medical complications and functional limitations), and a number of family variables including: sociodemographic factors, parental educational achievement, socio-emotional and physical functioning and family functioning. Lastly, we included retrospective self-reports of exposure to childhood physical and sexual abuse. The objective of this study was to examine the associations between individual and family-risk factors assessed in a sample of 4–16 year olds in 1983 and elevated BMI assessed in 2001 when they were young adults, at 21–35 years of age. The following issues were addressed. (1) What is the association between family contextual influences assessed in childhood/adolescence and BMI assessed in young adulthood? (2) Because of evidence that the influence of childhood risk factors may vary by gender, we also explored whether gender modifies the association between childhood risk factors on BMI in adulthood.

Typically, previous studies have used cross-sectional or retrospective reports of childhood risks to examine a limited number of factors. Few studies have examined the prospective relationship between multiple individual and family characteristics measured in childhood and BMI assessed in early adulthood [see [6,23,24,28]. Understanding childhood contextual influences operating at different levels is essential for determining which factors should be program targets for developing policies and early interventions to reduce obesity.

## Methods

### Sample

This study uses data from the initial (1983) and third (2001) waves of the Ontario Child Health Study (OCHS) - a prospective, longitudinal study of child and adolescent health in a cohort of 3,294 children ages 4–16 years, living in 1,869 households across Ontario, Canada [29]. The target population included all children born from January 1, 1966 through January 1, 1979, whose usual place of residence was a household in Ontario. A stratified, clustered, and random sample was selected from all household dwellings identified in the 1981 Census of Population. Sample weights were devised for the first wave based on the probabilities of selection and enlistment so that subject responses would be linked numerically back to the target population, improving the accuracy of statistical estimates. During a home interview, data were collected from parents (95% mothers) and adolescents aged 12–16 years by trained field staff from the Special Surveys Division of Statistics Canada. This study was approved by the Research Ethics Board at Hamilton Health Sciences, McMaster University.

### Variables and measures

#### Outcome variables

Information was collected from OCHS follow-up participants during a structured interview, administered in the home when participants were 21–35 years of age. BMI measured in 2001 was derived using self-reports of weight and height. Several studies have shown high correlation between self-reported and measured BMI [30-33]; however, self-reported BMI yields lower rates of obesity and overweight [34-36]. We calculated a corrected BMI [37] and ran all analyses on reported and corrected BMIs in parallel. We used correction equations based on the 2005 Canadian Community Health Survey (CCHS). These equations were generated using socio-demographic variables that were significantly associated with discrepancies between self-reported and measured values of BMI by sex. Because the results were identical for all models, we highlight findings based on the self-report BMI data only.

### Confounding variables

BMI has been linked to current income [6], education attainment [24] and physical and mental health [38,39]; therefore, these variables were included as potential confounders in all models. Our measures of potential confounders, measured in 2001, included: number of years of education (excluding grade repetition), household income in $1,000 s of dollars, and the SF-12® mental and physical health summary measures. The SF-12® is a valid and reliable standardized tool for assessing mental and physical functioning and overall health-related quality of life [40,41]. There are 12 questions, all selected from the SF-36®, which assess indicators of health, including: role limitation due to physical problems, general health perceptions, vitality, bodily pain, social function, role limitations due to emotional problems, and general mental health. These indicators are used to calculate two summary component scores: mental component score (MCS) and the physical component score (PCS). Lower scores indicate poorer levels of health functioning.

### Family variables

Maternal self-reports in 1983 provided key information on family variables including: (1) household income in 1,000 s of dollars; (2) receipt of social assistance (0 = *no*, 1 = *yes*); (3) both parents born outside of Canada (0 = *no*, 1 = *yes*); (4) average years of education for both parents in two-parent households or mother’s or father’s years of education in lone-parent households; 5) one or both parents with a functional limitation (0 = *no*, 1 = *yes*); (6) one or both parents with a chronic medical health problem (0 = *no*, 1 = *yes*); (7) one or both parents hospitalized for “nerves” or a nervous condition (0 = *no*, 1 = *yes*); and (8) one or both parents ever treated for “nerves” or a nervous condition (0 = *no*, 1 = *yes*). A single variable assessing family functioning was measured using the general functioning subscale of the McMaster Family Assessment Device (FAD) [42,43]. Statements described family behavior and relationships across six dimensions: problem solving, communication, roles, affective responsiveness, affective involvement, and behavioral control. Scale scores were summed and converted to *z* scores. The FAD has adequate one-week test-retest reliability (.66 to .76, depending on subscale), low correlations with social desirability scales (−.06 to -.19), moderate correlations with other self-report measures of family functioning (most expected correlations exceeded .50), and the FAD differentiates significantly between clinician-rated healthy and unhealthy families [43].

### Child variables

Child level variables include: gender (0 = *female*, 1 = *male*), age in years in 1983, as covariates, and low birth weight (0= *> 2500 grams*, 1 = *<2500 grams*) as a measure of prenatal risk. Four additional child risk variables were included for estimation of child health and functioning in 1983: (1) the presence or absence of a functional limitation (0 = *no*, 1 = *yes*); (2) the presence/absence of a medical condition (0 = *no*, 1 = *yes*); (3) the presence/absence of a psychiatric disorder (0 = *no*, 1 = *yes*); and (4) school performance. Mothers were the principal informants for measures of functional limitations and medical conditions. Measures of limitation of normal function and chronic illness or medical condition were adapted from various sources including the Rand Corporation’s Measure of Children’s Health and the Canada Health Survey [44]. Child functional limitation consisted of one or more limitations of normal functioning in physical activity (i.e. vigorous activity, bending, climbing), mobility (use of transportation and getting around the neighborhood), and self-care (daily activities—i.e. eating, dressing, bathing) due to illness, injury or medical condition, and/or limitation in role performance (kind or amount of ordinary play or schoolwork) due to physical, emotional or learning problems. Chronic illness or medical condition consisted of one or more illnesses/conditions present for at least 6 months’ duration derived from a list of 22 separate conditions [44]. Assessments of child psychiatric disorders via problem checklists were provided by mothers and teachers for children aged 4–11 years, and mothers and youths, for adolescents aged 12–16 years. Classification of child psychiatric disorder consisted of the presence of one or more conditions, including conduct disorder, emotional disorder, and attention-deficit disorder. The checklists were originally developed to screen for psychiatric disorder among children in the general population [29]. Our measure of school performance included both teacher and maternal assessment of school performance. The teacher assessment was based on a 4-item rating scale to the questions: “How would you describe the child’s current performance in the following categories: reading and English, spelling, arithmetic or math, and overall?” with response options 1 = *far below grade* to 5 = *far above grade*. Responses were summed and converted to *z* scores. Maternal assessments were used when teacher reports were missing and included the question: “Which of the following statements best describes how well ___ has done in school during the past 6 months”: ranging from 1 = *very well, excellent* to 5 = *not well at all, very poor student.* Responses were reverse coded and converted to *z* scores.

### Retrospective reports of childhood maltreatment

Most risk variables measured at the family and child levels were assessed in 1983; however, exposure to childhood physical and sexual abuse prior to the age of 16 years was assessed in 2001 using the Childhood Experiences of Violence Questionnaire Short-Form (CEVQ-SF) [45]. The CEVQ-SF is a brief, reliable, valid, retrospective self-report measure assessing exposure to victimization and maltreatment [45,46]. Assessment of child physical abuse (PA) consists of 3 items assessing the frequency with which the individual was exposed to: (i) an adult slapping their head or face, or spanking with an object, (ii) having something thrown at them or being shoved, and (iii) being physically attacked, burned, choked or punched before age 16. Assessment for exposure to child sexual abuse (SA) consisted of a single question, “before age 16 when you were growing up, how many times did an adult ever do any of the following things when you didn't want them to: touch the private parts of your body or make you touch their private parts, threaten or try to have sex with you or sexually force themselves on you?” Each item consisted of a 5-point response option ranging from 1 (*never*) to 5 (*10+ times)*. The cut-off score for severe physical abuse (PA) exposure was > 10 times for the first two PA items (score of 5), and 1–2 times (score of 2 or above) for the third PA question. The cut-off for sexual abuse exposure was a response of 1–2 times (score of 2 or above) on the SA question. If a respondent met the cut-off criteria for severe PA or SA, they were coded as 1. The two-week test reliabilities of the CEVQ-SF for measuring PA, severe PA, and SA in an earlier study were: κ = 0.61, κ = 0.72 and κ = 0.91 respectively [46].

### Multiple imputation and attrition weights

Overall, 1,928 (58.5%) of the original 3,294 children were complete respondents in 2001 and an additional 427 (18.1%) completed abbreviated interviews; 910 were non-respondents, and 29 were excluded due to death (*n* = 26) or institutional placement (*n* = 3). The final sample for analysis included 1,928 participants. There were 549/1,928 (28.5%) participants with missing values on individual variables (i.e. 256 on only one variable and 293 on two or more variables). To estimate values for missed responses, we used multiple imputation in SPSS 19.0 to create five complete data sets. Missing family variables were imputed at the family level, whereas child variables were imputed at the child level. Models from each of these data sets were individually run in MLwiN 2.24 [47] and parameter estimates and the estimated standard errors (SEs) were combined using Rubin’s rules [48].

Attrition weights were developed and applied to the original 1983 sample weights to recapture the original sample characteristics [28,49] using weighted complete-case analysis [50]. Fourteen variables measured in 1983 were selected to model non-response in 2001. The 1983 variables included were: child health status, functioning, and health service use; measures of parental health, family structure and functioning; and numerous indicators of family socioeconomic disadvantage. Several selected variables were associated with attrition, including child use of mental-health social services: 3.1% respondents, 7.5% no respondents (4.4% in 1983); and family in rental housing; 17.6% respondents, and 30.2% no respondents (21.3% in 1983).

To test the accuracy of the attrition weights, baseline characteristics of the OCHS sample in 1983 were compared with estimates derived using attrition weights applied to respondents at follow-up in 2001. This comparison yielded very similar estimates; for example, on the two variables identified above, this comparison yielded 4.4% versus 4.0% for child utilization of mental health-social services, and 21.3% versus 21.4% for family in rental housing. Health and functioning of OCHS respondents as young adults in 2001 were also compared with an independent probability sample of age-matched peers (N = 5,718) living in Ontario and participating in the CCHS (Statistics Canada, 2004). Weighted estimates on identical variables derived from OCHS versus the CCHS were very close, for example, male sex (51.7, 50.1%), at work last week (80.6, 80.3%), personal income < $15,000 (21.6, 22.9%), excellent health (32.6, 32.3%), has asthma (11.4, 11.5%), smokes daily or occasionally (34.3, 35.2%) [49].

### Statistical analysis

The information collected on children in this study form a hierarchical structure consisting of individual children (Level 1, or child level) nested in families (Level 2, or family level). In this study, we use multilevel linear regression and the statistical software MLwiN 2.24 [47] to estimate the extent to which BMI assessed in 2001 is associated with child and family level risk factors assessed in 1983. In multilevel modeling, residual error is partitioned across levels, thereby capturing the extent to which variation in response is associated with each level. Fixed effect estimates in regression using linear multilevel modeling are interpreted in the same fashion as fixed effects in ordinary least squares regression: the intercept is the estimated mean response and the beta coefficients denote an increase or decrease in the dependent variable associated with one unit of change in each of the independent variables. Our modeling strategy consisted of introducing all confounding variables, including the 2001 variables (current education, income and mental and physical health scores), and child gender and age in 1983, as confounding factors, followed by family risk indicators (Model 1). This was then followed by the introduction of the child variables (Model 2). To examine whether there was a differential effect based on the age of the child in 1983, we initially stratified by childhood age, childhood (4–11 years) and adolescence (12–16 years). Because the results were consistently similar across age categories, we only report findings on the entire sample.

## Results

Table1 presents the sample characteristics, including the percent of families (n = 1,270) and children (n = 1,928) classified by each contextual variable, along with BMI in 2001. In young adulthood, the mean level of BMI was 25.38 (Table1). Using cut-offs categorized by the World Health Organization, 2.5% of the sample were underweight (BMI < 18.5 kg/m^2^), 51.2% were normal weight (BMI > 18.5 and < 25 kg/m^2^), 31.1% of the sample were overweight (BMI ≥ 25 kg/m^2^) and 15.2% were obese (BMI ≥30 kg/m^2^). These findings are comparable to prevalence rates in self-reported BMI data in other Canadian studies [51,52].

**Table 1:** Sample Characteristics

**Characteristic**	
Families, *N* = 1,270	
Family income in $1,000 s (*M, SD*)	32.37, 15.50
Receipt of Social Assistance (*n*)	5.5% (106)
Both parents born > Canada(*n*)	19.5% (375)
Parent education in years (*M, SD*)	11.93, 3.38
Parent medical health problem (*n*)	21.2% (409)
Parent functional limitation (*n*)	8.2% (159)
Parent treated for “nerves” (*n*)	16.2% (311)
Parent hospitalized for “nerves” (*n*)	5.9% (119)
Family Functioning (*M, SD*)	36.14, 5.20
Children, *N* = 1,928	
*Child health in 1983*	
Male child (*n*)	48.8% (941)
Age (*M*, *SD*)	10.08, 3.68
Medical condition (*n*)	16.2% (309)
Functional limitation (*n*)	4.5% (85)
Psychiatric disorder (*n*)	10.2 (197)
School performance--- teacher/parent (*M*, *SD*)	3.36, 0.95
Low birth weight (*n*)	2.7% (53)
*Retrospective Assessment in 2001 of Childhood Abuse*	
Severe Physical Abuse (*n*)	18.2% (351)
Sexual Abuse (*n*)	5.3% (107)
*Outcomes and Covariates in 2001*	
Body Mass Index (*M*, *SD*)	25.38, 4.78
Education in 2001	15.15, 2.70
Income in 2001 in $1,000 s (*M, SD*)	33.79, 21.97
SF-12® Mental Component Score (*M, SD*)	18.23, 2.11
SF-12® Physical Component Score (*M, SD*)	22.32, 3.32

### Influence of family and child variables on BMI

Variability in BMI attributable to family-level differences is estimated by the intraclass correlation coefficient (ICC). The ICC is derived from the random effects variances reported in the multilevel null model and represents the total unexplained variance in BMI associated with between-family differences: 39.05% from 8.92/(8.92 + 13.92) (not shown).

Multilevel regressions for BMI are presented in Table2. Controlling for current education, income and mental and physical health status, and for child age and sex, we found that in Model 1 (Family Variables), individuals from families who received social assistance during their childhood had higher BMI in early adulthood (2.02, *p* < 0.001), while being the child of immigrant parents (born outside of Canada) was associated with lower BMI (−1.20, *p* < 0.001). Family income was not significantly associated with BMI (.004). In addition, parental education (positive), parent hospitalized for “nerves” and family functioning (negative) exhibited significant associations with BMI. Every one year increase in parental education was associated with 0.18 decrease in BMI (*p* < 0.001), whereas having a parent hospitalized for nerves was associated with increases in BMI of 1.20 (*p* < 0.05).

**Table 2:** Multilevel Models Neighborhood, Family, and Childhood Influences on BMI (b and (95% Confidence Interval))

	**Model 1 Family Variables**	**Model 2 Child Variables**
Fixed effects		
Intercept	24.76 (24.35 to 25.16)	25.62 (24.64 to 26.59)
Family Variables		
Family income in $1,000 s	0.004 (−0.01 to 0.02)	0.0038 (−0.01 to 0.02)
Social assistance	2.02 (0.92 to 3.11)***	1.89 (0.26 to 3.52)*
Immigrant parents	−1.20 (−1.78 to −0.61)***	−1.20 (−1.73 to −0.66)***
Education in years	−0.18 (−0.25 to −0.09)***	−0.17 (−0.26 to −0.09)***
Parent medical problem	0.47 (−0.12 to 1.06)	0.46 (−0.13 to 1.06)
Parent functional limitation	−0.50 (−1.35 to 0.36)	−0.50 (−1.40 to 0.37)
Parent treated for “nerves”	−0.10 (−0.74 to 0.55)	−0.16 (−0.85 to 0.53)
Parent hospitalized for “nerves”	1.20 (0.15 to 2.24)*	1.19 (−0.01 to 2.39)
Family functioning	−0.05 (−0.10 to −0.007)*	−0.06 (−0.11 to −0.01)*
Child variables		
Age in years	0.11 (0.05to −0.22)**	0.10 (0.04 to 0.17)**
Male	1.28 (0.87 to 1.70)***	1.27 (0.66 to1.59)***
Psychiatric disorder		1.12 (0.31 to 1.94)**
Functional limitation		−0.11 (−1.43 to 1.21)
Medical condition		−0.03 (−0.71 to 0.66)
School performance		−0.26 (−0.50 to −0.01)*
Low birth weight		−0.77 (−2.19 to 0.64)
Physical abuse		0.12 (−0.47 to 0.72)
Sexual abuse		0.50 (−1.33 to 0.64)
Random Effects (*SE*)		
Level 2, Family	7.65 (0.79)	7.60 (1.27)
Level 1, Child	13.03 (0.64)	12.87 (0.99)
−2*log likelihood	11,519	11,498

* p < .05, ** *p* < .01, ***p < .001.

In Model 2 (Child Variables), individuals classified with a childhood psychiatric disorder and poorer school performance exhibited higher BMI (*p* < 0.01 and 0.05, respectively). Low birth weight status was not significantly associated with BMI. The total proportional reduction in error (explained variance) associated with the predictor variables in Model 2 is 1-(7.60 + 12.87)/(8.92 + 13.92) or 10.4%.

**Figure 1:**
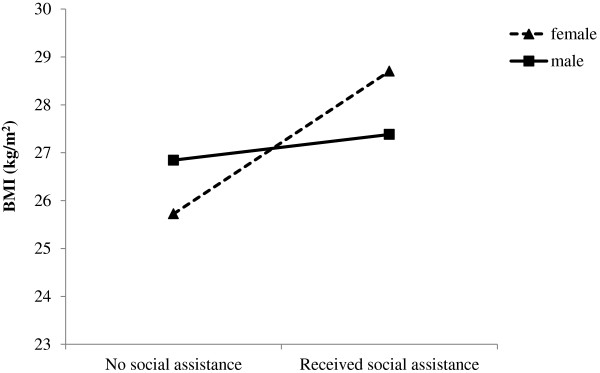
Gender moderates the association between receipt of social assistance in childhood/adolescence and BMI in early adulthood.

**Figure 2:**
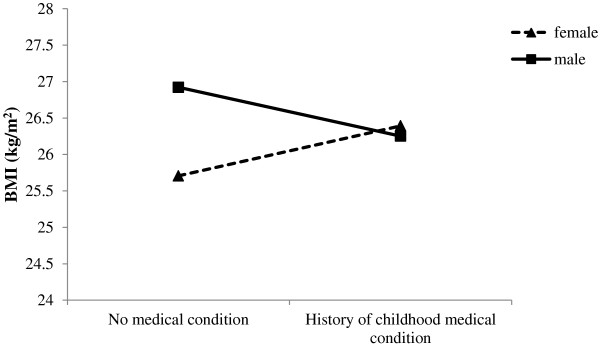
Gender moderates the association between presence of a medical condition in childhood/adolescence and BMI in early adulthood.

To explore if associations between family and child variables and adult BMI were modified by gender, we tested statistical interactions between gender and all of the variables in Model 2 in Table2. Each interaction was tested on its own (i.e., added separately to Model 2). There were two statistically significant interactions: one involving gender by receipt of social assistance (β = −2.44, *SE =* 1.05) and the other involving gender by childhood history of medical condition (β = −1.36, *SE = .*58). As shown in Figure1, there was a positive association between receipt of social assistance and BMI for females but not for males. Similarly, as illustrated in Figure2, females with a childhood history of a medical condition had higher BMIs than those with no history of a medical condition. This effect was not seen in males. As mentioned above, we replicated these findings using corrected BMI (data not shown).

## Discussion

Using a comprehensive model, simultaneously incorporating child and family variables in a prospective design, we examined associations between a number of risk factors experienced in childhood/adolescence and BMI in early adulthood, adjusting for respondents’ age, sex, education, income and health. This study provides several important findings at the family level: 1) socioeconomic adversity (measured by receipt of social assistance) was related to increased BMI, whereas parental education was associated with lower BMI in early adulthood; 2) parental immigrant status was associated with lower BMI; 3) family functioning was negatively related to BMI (higher family function was associated with lower BMIs) and 4) parental mental health problems were associated with increased BMI. At the child level, presence of child mental disorders and poor school performance were both related to higher BMI, even after controlling for current education and mental health status. Altogether, the predictor variables explained about 10% of respondent variability in BMI at young adulthood. In the fully adjusted model, the effects of variables such as immigrant status and social assistance converted to standard deviation units exhibited what would be considered small to medium effects (*d =* 0.25 and 0.40) based on Cohen’s criteria [53].

In this study, we found that 39.05% of the variation in BMI was associated with between-family differences. The familial aggregation of BMI reflects a multifaceted interplay between genetic susceptibility to weight gain and shared environmental influences within families. Some of these shared influences are linked to measured family risk factors, such as socioeconomic disadvantage, parental education and family functioning. Recent studies have found that genetics play an increasingly important role in explaining variation in BMI over time, with its greatest influence in late adolescence; whereas environmental influences decrease over time, exerting their strongest effects early in childhood and adolescence [54-56].

Our finding that family status factors (parental education and receipt of social assistance) are associated with BMI is consistent with previous research [5-8,10]. In children and adolescents, lower SES, regardless of how it is measured (parental education, occupation, or income), is associated with increased BMI and obesity [57-59]. Economic disadvantage in families may be directly related to a number of factors that pose a risk for higher BMI, such as engaging in less physical activity, poorer nutrition and eating habits, and lack of participation in organized sports [60,61]. In addition, early in childhood, healthy lifestyle trajectories may be set via modeling by caregivers [61]. We also found that parental immigrant status was negatively related to BMI in adulthood. This finding is in agreement with a recent report indicating that first generation immigrants have lower BMI compared to second generation or Canadian born children [62]. Interestingly, children from that study lived in a multi-ethnic, disadvantaged inner city community, where many immigrants initially settle. These areas are typically characterized by lower levels of education and income. Despite these socioeconomic risks, immigrant status still conferred a protective influence in this sample; however, this protective health advantage may be lost over time with exposure to unhealthy lifestyle habits in host communities.

Family functioning was associated with lower BMI in early adulthood. Our measure of family functioning was comprised of questions on problem solving, affective responsiveness and involvement and behavioral control within the family. These questions tap into parenting practices. Research investigating the impact of parenting practices on BMI in children and adolescents has found that authoritative homes, characterized by a family context of warmth, high emotional support, encouragement, monitoring and bidirectional communication, is related to healthier eating habits, increased physical activity and lower BMI [63-67]. Interestingly, our distal family status factors (parental education and receipt of social assistance) remained significant even after adding family functioning to the model. This implies that family functioning does not act as a mediator between family status and BMI but exerts its own independent effects. Pathways relating family functioning to BMI are likely complex and may impact weight through its direct influence on diet and physical activity [66] or through more indirect mechanisms such as child self-regulation capabilities [68].

Parental mental health problems predicted elevated BMI, although this association became non-significant when childhood variables were added to the model. Consistent with previous findings [15,19,27], we found that childhood psychiatric disorder and school difficulties were associated with greater BMI in early adulthood, even after controlling for current mental health status and years of education. Although there is some debate regarding the magnitude of the effect, starting at an early age [15] mental health problems are established predictors of elevated BMI through the lifespan [16-18]. Given the established nature of this association, there is likely an underlying mechanism shared by both mental health and obesity. Potential candidates include genetic, behavioral, and/or psychological factors which are common to both phenotypes [27,69]. Contrary to previous research [21,22], we did not find an association between history of childhood physical or sexual abuse and adult BMI. This may be due to the fact that we have such a comprehensive array of risk factors measured. Also contrary to previous research [12,13], we did not find an association between low birth weight and adult BMI. This may be due to reporting inaccuracies: birth weights were based on self-reports by mothers several years after the birth of their child. The prevalence of low birth weight in this sample (2.5%) is lower than prevalence rates reported for Ontario (4.8%), suggesting that low birth weight may be under represented in our sample.

We found that gender moderated the effect of two risk factors on BMI: receipt of social assistance and presence of a medical condition in childhood. In females, but not in males, the presence of these risk factors was associated with higher BMI in early adulthood. Our results support other findings that childhood socioeconomic disadvantage is associated with later obesity in women [70-72]. The association between BMI and later chronic disease in adulthood is fairly well characterized; however research linking medical problems in childhood to later BMI is relatively scarce. One recent study found that childhood leukemia was associated with increased BMI in adulthood but only in females [73]. Presence of medical conditions or chronic illnesses in childhood may place greater limitations on physical activity, leading to a more sedentary lifestyle. In addition, it is noteworthy that within the OCHS, the prevalence of a chronic illness or medical condition in 1983 was not evenly distributed across socioeconomic groups. Children from low income households, defined as below the Statistics Canada poverty line, had higher rates of medical conditions compared to children of families above the poverty line [50]. It is possible that these gender differences are linked to variations in underlying biological mechanisms, such as HPA axis function given that there are sex differences in stress system structure and function [74] and that the HPA is linked to BMI [75,76], various medical conditions [77] and disparities in SES [78]. Theoretically, it is argued that females have a heightened predisposition to stress-related disease with exaggerated sensitivity to stress which pushes females over the “disease threshold” [79]. If this is true, medical conditions or socioeconomic disadvantage experienced in childhood may lead to biological sensitivities to stress, especially in females, putting them at greater risk of elevated BMI later in life.

### Limitations

Despite strengths of the OCHS in assessing childhood risks associated with BMI in early adulthood, this study has limitations. First, approximately 30% of 1983 participants were lost over the 18-year follow-up. Because this loss was selective to socioeconomic disadvantage, Boyle and colleagues [49] devised attrition weights that integrated original sample selection probabilities from 1983. We believe that any potential systematic bias is likely to be small and would be more related to underestimation of the influence of risk factors on outcomes. Second, there are a few measurement limitations. All risk factors were measured at one point in time, and we are not able disentangle their temporal associations or to assess intervening variables between 1983 and 2001. Our assessment of risk factors was collected prospectively over twenty years ago. Despite this lapse of time, we believe our findings are currently applicable to young adults who are likely to experience the same risk factors. We are unaware of any changes that would mitigate the association between childhood risk factors and BMI, as found in this study. Two, we do not have measures of child or parent BMI in 1983. It is well established in studies tracking weight status in children that those with higher BMIs early on tend to maintain these trajectories throughout adolescence and into adulthood, indicating some stability for most individuals [11,80,81]. Our measure of BMI was also based on self-report. Self-reported BMI yields lower rates of obesity and overweight [35-37]. Nevertheless, self-reported BMI remains an important tool for health surveillance [51]; commonly used because it is a simple, economical, and non-invasive method of collecting data from large samples [82]. Moreover, self-reported BMIs are related to morbidity and mortality [83-85]. Applying a BMI correction factor did not alter our findings. Three, our abuse risk indicators were measured retrospectively. In general population studies, such assessments are very difficult to obtain from children prospectively and there is no reason to believe that current BMI would influence the recall of these experiences. Fourth, in exploring interactions between gender and childhood risk factors, we are vulnerable to obtaining a significant effect because of multiple testing. We chose to explore these interactions because of the lack of consistent research evidence on this question and the recognition that differential weight and body fat composition are integral to gender differences. 

## Conclusions

In conclusion, this study provides support for the notion that a significant proportion of the variation in BMI is attributable to family factors. More studies are needed to disentangle the influence of specific family factors on BMI, including genetic by environment interactions. To date, most preventive programs for childhood obesity are school-based and focus, with limited effectiveness, on healthy-eating, active living, and mental well-being initiatives [86-88]. Our findings suggest that preventive interventions and policy practices need to target family environments early in childhood, especially environments where multiple risk indicators are present in the family. Family based programs targeting parenting styles and skills, as well as dietary behaviors and physical activity have demonstrated positive effects on children’s weight loss [89,90]. More research and development of family-based prevention and treatment programs is needed. 

## Competing interests

None of the authors have any financial or conflict of interest to declare.

## Authors’ contributions

Author contributions include the following: AG was responsible for conception and design, analysis and interpretation of data, and drafting the article. LA, LD and HLM were responsible for conception and design of data, and critical revision of paper for important intellectual content. MB and KG were responsible for statistical analyses and interpretation of data, and revising the article critically for important statistical and intellectual content. All authors had full access to all of the data (including statistical reports and tables) in the study, can take responsibility for the integrity of the data and the accuracy of the data analysis, and approved the final version to be published. In collaboration with Statistics Canada, MB was responsible for the design and implementation of the study.

## Funding

The 1983 research funding was provided by the Ontario Ministry of Community and Social Services. The follow up in 2001 was funded by a grant from the Canadian Institutes of Health Research (CIHR) awarded to Dr. Boyle. Dr. Gonzalez was funded by a Canadian Institutes of Health Research (CIHR) Postdoctoral Fellowship from the Institute of Gender and Health and a Lawson Postdoctoral Fellowship. Dr. Boyle received support from a Canada Research Chair from CIHR in Social Determinants of Child Health. Dr. MacMillan received support from the David R. (Dan) Offord Chair in Child Studies.

Neither funding agency had direct involvement in the design and conduct of the study; in collection, management, analysis, and interpretation of the data; or in preparation, review, or approval of the manuscript.

## Pre-publication history

The pre-publication history for this paper can be accessed here:

http://www.biomedcentral.com/1471-2458/12/755/prepub
